# Uneven primary healthcare supply of rural doctors and medical equipment in remote China: community impact and the moderating effect of policy intervention

**DOI:** 10.1186/s12939-024-02183-7

**Published:** 2024-05-13

**Authors:** Lu Shan, Yingying Gan, Xiang Yan, Shuping Wang, Yue Yin, Xiaofan Wu

**Affiliations:** 1 Department of Urban Planning and Design, The University of Hong Kong, Hong Kong, SAR China; 2 The Faculty of Architecture and Building Environment, Delft University of Technology, Delft, The Netherlands; 3 College of Architecture and Environment, and the Institute of Urbanization Strategy and Architecture Research, Sichuan University, Chengdu, Sichuan province China; 4 China National Health Development Research Center, Beijing, China; 5 Chinese Medical Doctor Association, Beijing, China; 6 Peking University First Hospital, Beijing, China

**Keywords:** Primary healthcare (PHC), Service capacity, Community environment, Multilevel model, Difference in difference model, Remote rural area, China

## Abstract

**Background:**

Unequal access to primary healthcare (PHC) has become a critical issue in global health inequalities, requiring governments to implement policies tailored to communities’ needs and abilities. However, the place-based facility dimension of PHCs is oversimplified in current healthcare literature, and formulating the equity-oriented PHC spatial planning remains challenging without understanding the multiple impacts of community socio-spatial dynamics, particularly in remote areas. This study aims to push the boundary of PHC studies one step further by presenting a nuanced and dynamic understanding of the impact of community environments on the uneven primary healthcare supply.

**Methods:**

Focusing on Shuicheng, a remote rural area in southwestern China, multiple data are included in this village-based study, i.e., the facility-level healthcare statistics data (2016–2019), the statistical yearbooks, WorldPop, and Chinese GDP’s spatial distribution data. We evaluate villages’ PHC service capacity using the number of doctors and essential equipment per capita, which are the major components of China’s PHC delivery. The indicators describing community environments are selected based on extant literature and China’s planning paradigms, including town- and village-level factors. Gini coefficients and local spatial autocorrelation analysis are used to present the divergences of PHC capacity, and multilevel regression model and (heterogeneous) difference in difference model are used to examine the driving role of community environments and the dynamics under the policy intervention.

**Results:**

Despite the general improvement, PHC inequalities remain significant in remote rural areas. The village’s location, aging, topography, ethnic autonomy, and economic conditions significantly influence village-level PHC capacity, while demographic characteristics and healthcare delivery at the town level are also important. Although it may improve the hardware setting in village clinics (coef. = 0.350), the recent equity-oriented policy attempts may accelerate the loss of rural doctors (coef. = − 0.517). Notably, the associations between PHC and community environments are affected inconsistently by this round of policy intervention. The town healthcare centers with higher inpatient service capacity (coef. = − 0.514) and more licensed doctors (coef. = − 0.587) and nurses (coef. = − 0.344) may indicate more detrimental policy effects that reduced the number of rural doctors, while the centers with more professional equipment (coef. = 0.504) and nurses (coef. = 0.184) are beneficial for the improvement of hardware setting in clinics.

**Conclusions:**

The findings suggest that the PHC inequalities are increasingly a result of joint social, economic, and institutional forces in recent years, underlining the increased complexity of the PHC resource allocation mechanism. Therefore, we claim the necessity to incorporate a broader understanding of community orientation in PHC delivery, particularly the interdisciplinary knowledge of the spatial lens of community, to support its sustainable development. Our findings also provide timely policy insights for ongoing primary healthcare reform in China.

## Introduction

Since it was first conceptualized by the World Health Organization [1], primary healthcare (PHC) has become an important form of healthcare for achieving universal health coverage [2]. Unlike traditional healthcare models (e.g., hospital), PHC is often envisioned as “a way to overcome health disparities operating at numerous scales through the provision of equitable and accessible care by, in, and for communities ([3], p270)”. Jointly affected by the increasing burden of non-communicable chronic diseases (NCDs) and the scarcity of healthcare resources, the primary healthcare sector is now under increasing pressure to deliver PHC more efficiently to enable more population benefit from the PHC agenda [4–7].

Against this background, an essential consensus has been reached that the spatiotemporal dynamics of the allocation of PHC resources are closely associated with the local community environments, emphasizing the social determinants of healthcare inequalities [8]. Community is a fundamental geographical concept that has been repeatedly reinterpreted in extent literature, e.g., the “natural place” by the Chicago School [9, 10] and the “social area” by the social organization lens [11], with most definitions involving two general components: physical and social. Scholars and policymakers commonly herald that the basic elements of communities, such as population composition, geographical landscape, and territorial characteristics, are organized in a social system to constitute community environments and changes, in terms of the relationship between residents and places and among various residents [12, 13].

**Fig. 1:**
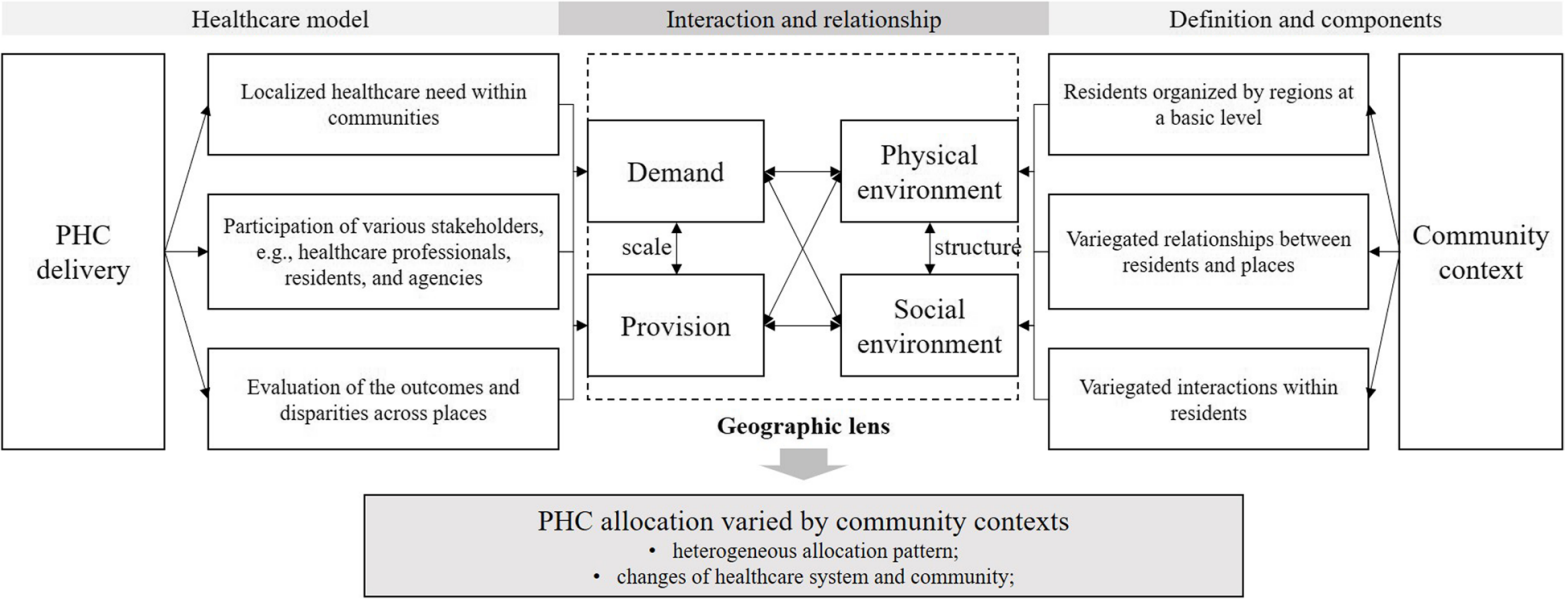
The interrelationship between PHC allocation and community environments

The extant literature provides an essential basis for examining the embeddedness of PHC in the community, particularly in three ways that are shown in Fig. 1. First, PHC services must be responsive to the highly localized needs of community residents, highlighting that PHC’s priorities are delineated to the local community, rather than to a national or regional context [14]. Second, PHCs are configured based on the locations, collaborating agencies, and stakeholders in the community [15]. For example, a Delphi study identified four community-oriented operational dimensions of PHCs: client/community engagement, equity, intersectoral team practices, and demographic orientation [16]. Third, the community serves as the fundamental spatial unit for assessing the equity of PHC, although these policies are often outlined at the regional level. The critical point is to understand how PHC practices interact at different administrative scales and the bottom-up features reflected in PHC delivery, e.g., professional’s preference for career opportunities (X [17, 18].). To this extent, the community lens provides a grassroots way to understand how PHC resources are allocated and the related inequalities, as stated in the Almaty Declaration: ‘[PHC] reflects and evolves from the economic conditions and sociocultural and political characteristics of the country and community.’

However, questions about the uneven supply of PHC resources and the mechanism associated with community environments have yet to be thoroughly answered. In healthcare research, although some prior research has involved socioeconomic factors such as rurality, population density and income levels (Soares [19, 20]), the established indicator system for quantifying community attributes needs a more comprehensive understanding of the spatial dimension. For health geographers, selecting appropriate proxies for measuring PHC services presents difficulties and existing literature mainly focuses on the locational characterization of PHCs [21, 22, 23]. Importantly, few studies have been focused on remote rural areas, especially in developing contexts. The examination in the disadvantaged places may direct more attention toward the significance of the uneven distribution of PHC resources and provide more efficient and valuable implications for policymakers.

Addressing the abovementioned gap is critical in China’s public-dominated PHC delivery, given the increasing PHC capacity disparities by locales [24]. In China, primary healthcare facilities align with a hierarchical structure comprising three administrative levels: county/district, township/sub-district, and village/residential committee. Villages refer to the most basic settlements with populations of fewer than 3000. Several proximate villages collectively form a township or town, and aggregations of proximate towns constitute counties, with populations ranging from 100,000 to 1 million residents. There is commonly one township healthcare center per town and one clinic per village in China, with clinics at a lower level and operating more independently [25]. Notably, while township healthcare centers play a crucial role in facilitating access to healthcare resources at the village level through various means, these two types of PHC institutions are de facto independent entities within the PHC system, with potential conflicts such as competition for healthcare resources and market share  [26, 27].

Such mixed relationships between township healthcare centers and village clinics have been even more complex since the 2009 PHC reform, due to the differentiated policy priority [26]. To address the widespread problem, e.g., fragmentation of healthcare services, recent years has seen heightened government efforts in PHC system across various scales in China, particularly the promotion of medical alliances [28]. However, the town- and village-level institutions have developed to varying degrees because local governments commonly regard the township healthcare centers as the policy priority (Y [26].). To this extent, there remains a limited understanding of the policy intervention ramifications on village-level PHCs, and a need remains for additional investigation to assess the village-level PHCs’ service capacity from the supply side, especially regarding the role of township healthcare delivery.

Drawing official statistical data from Shuicheng County, a remote rural area in southwestern China, this study aims to examine the correlations between community environments and the spatial inequalities of PHC and the moderating effects of recent policy intervention. Corresponding to the claims of the state, Shuicheng County has issued various but interrelated policies recently to enhance the capacity of grassroots healthcare services, including (1) unified management of village clinics’ finances; (2) optimizing the hardware facilities at various medical institutions; (3) integrated management of health services within communities/villages; (4) encouraging the mobility of rural doctors within higher and lower-level institutions [29–31]. Therefore, examining the uneven PHC supply and the correlations with local communities in Shuicheng can provide not only a nuanced understanding of the supply-side mechanism of the uneven PHC delivery but also timely policy implications for ongoing equity-oriented PHC reform in rural China.

The remainder of the paper is organized as follows: section 2 introduces the study area, data, and methodology; section 3 reports and interprets the results while section 4 discusses; section 5 is about the conclusions and limitations.

## Methodology

### Study area and data source

**Fig. 2:**
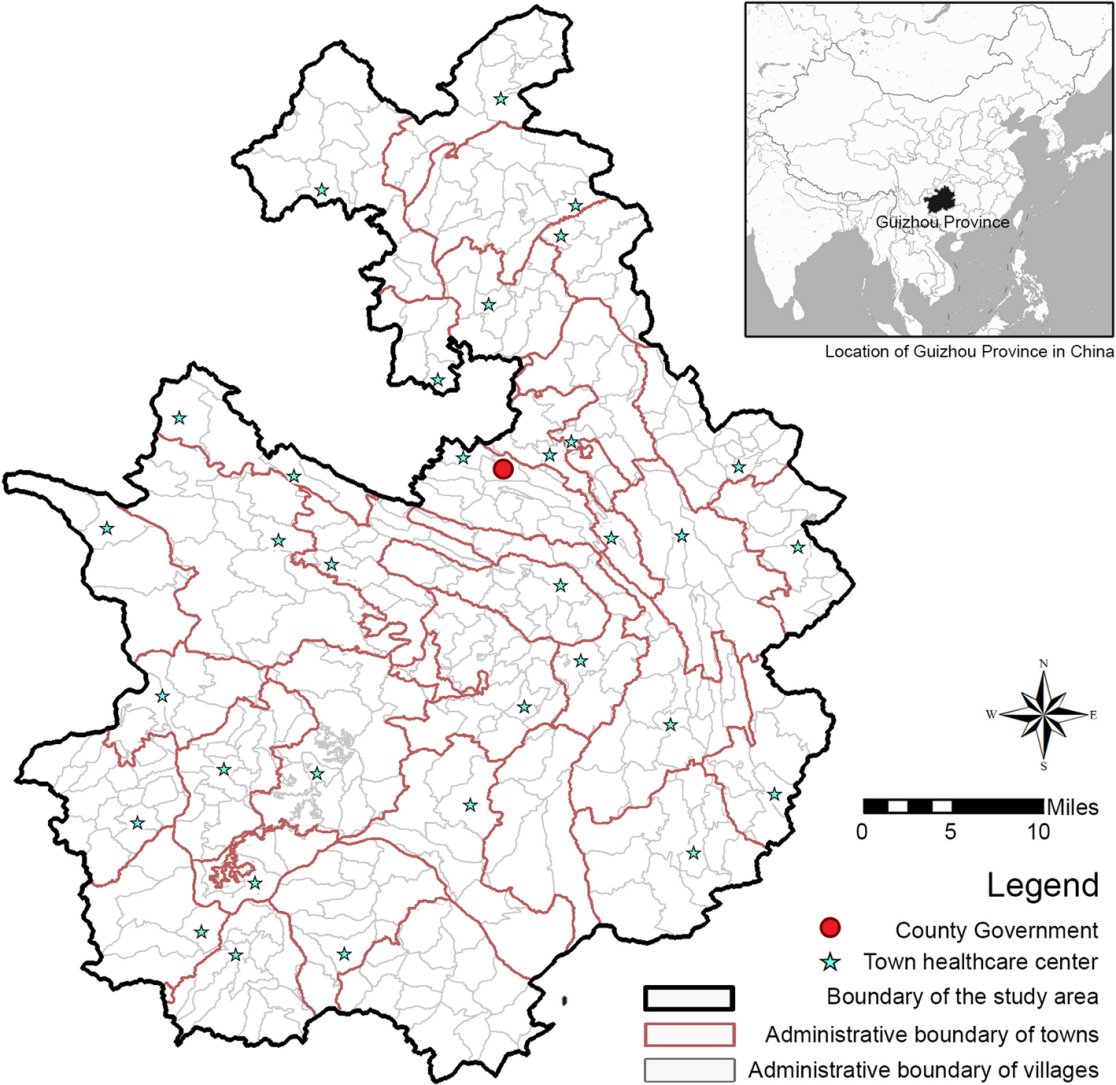
The study area

This study is conducted in Shuicheng County (Fig. 2), located in Guizhou Province, which has been designated as a national-level pilot area for the comprehensive prevention and control of chronic diseases. As of the end of 2019, Shuicheng County had a registered population of 760,500. It is administratively divided into 30 townships (towns, sub-districts), comprising 4 sub-districts, 13 towns, and 13 townships, with the latter including 5 northern townships such as Mugu, Baohua, Nankai, Jinpen, and Qinglin. The trend of decreasing population density with increasing distance from the county seat is observed in the study area. Specifically, Shuangshui Street, which was the original location of the county government, constitutes only 6.46% of the total county population. This highlights the sparsely populated nature of the area, which can be classified as relatively remote within China. As of 2021, the county comprises a total of 229 medical and health institutions at various levels, including 3 county-level hospitals, 30 township medical centers, and 181 village clinics. The county also accommodates 17 privately-run hospitals. On average, each village clinic is staffed by 2 to 6 medical personnel, catering to an average resident population of 3013 individuals. The county’s medical institutions collectively possess 3525 beds and employ 3123 healthcare personnel.

Shuicheng serves as a proper case for this study for three main reasons. First, the mountainous terrain and poor transportation infrastructure in Shuicheng lead to a high dependence of villagers on villages, making it an ideal case for understanding the PHC landscape in rural China. Second, the relatively low per capita GDP of Shuicheng (ranks 22nd out of 88 counties in Guizhou Province) makes it better illuminate the wide breadth of PHC disparities within rural China, particularly in remote regions with limited resources. Third, Shuicheng implemented a county-level medical alliance to restructure its PHC system for all towns and villages. In concrete implementation, in 2018, four townships and their jurisdictional villages, namely Bide (比德), Panglong (蟠龙), Miluo (米箩) and Faer (发耳), were the first to complete the reform [32]. Till the end of the observed periods in this study, that is, at the end of 2019, all townships in ShuiCheng County have completed the construction of “county-level medical alliance” [33]. Therefore, this offers a unique opportunity to examine the dynamic relationship between PHC inequalities and communities by revealing the intervention impact of China’s recent healthcare reform.

We conduct a dataset consisting of official statistics and open data in this study. The facility-level healthcare information is extracted from the *Shuicheng County Healthcare Statistic Annual Report*, which is accessed by one of the authors in collaboration with the local Health Commission. This official report provides detailed and reliable information about Shuicheng’s primary healthcare system between 2016 and 2019. The socioeconomic information at the town level is obtained from the official statistical yearbooks during the same time periods. The demographic information of administrative villages is accessed from WorldPop (https://www.worldpop.org/), an open platform that provides annual demographic data from dozens of countries worldwide and has been widely used in relevant works in recent years [34, 35, 36]. To ensure accuracy, we selected the 100 m*100 m raster data and then fit it to the administrative boundaries of villages. The village-level economic information is obtained by integrating the Statistical Yearbook of Shuicheng County and Chinese GDP’s spatial distribution network data provided by the Resource and Environment Science and Data Center [37]. The data processing is mainly performed using ArcMap software.

### Outcomes: village-level PHC service capacity

Echoing the concerns in existing literature [38, 39], this study utilizes rural doctors and medical equipment to evaluate PHC capacity in each village, i.e., the outcomes in this study. Rural doctor refers to the number of doctors per capita who are registered to practice in each village, including four specific types (i.e., western medicine, traditional Chinese medicine, integration of traditional Chinese and western medicine, and ethnic medicine). Medical equipment is measured by calculating the number of 12 types of essential medical equipment per capita (shown in the Appendix), such as stethoscopes, sphygmomanometers, and respirators, which are regulated by *the Village Clinic Service Capacity Standard (2022)* in China. Particularly, owing to the scarcity of nursing personnel in China’s remote rural areas and the resulting data availability in Shuicheng, the number of nurses in each village clinic is not included in this study, although it is an important indicator of PHC services.

### Exposure: community environments and policy intervention

Based on the embeddedness of PHC in community environments analyzed previously and the data availability in China, this study measures the community environments of villages as the exposures at two levels: the village level and the town level. To reflect the broad socio-spatial factors that shape localized healthcare needs, for village level, the ethnic composition, the proportion of young people (less than 20 years old) and the elderly (over 65 years old) are used to describe the demographic influence; the gross output value of agriculture and industry and the spatial distance to the county government is used for the economic influence; whether the village belongs to a mountainous area is used to measure the locational influence. In contrast, given China’s PHC contexts, the other two perspectives are combined and examined at the town level. The provision of high-quality medical equipment, the actual number of healthcare beds, licensed doctors, and nurses in township healthcare centers are used to measure the interaction between various healthcare agencies, while the township population, aging degree, industry structure, and public financial revenue are used to assess the administrative division features across place. The year of records is used to control for time-fixed effect. In addition, the difference in difference model includes a dummy variable to identify whether the township healthcare center completed the construction of the county medical alliance, serving as another important exposure in this study. Table 1 illustrates variable definitions and data descriptions.

**Table 1:** Variable definitions and data descriptions

Variable	Definition	Mean (%)	Std. Dev.
**Dependent variable**			
Rural doctor	The number of rural doctors per capita in each village.	0.04	0.03
Medical equipment	The number of essential medical equipment per capita in each village.	0.22	0.15
**Village level**			
Ethnic autonomous	Dummy variable, determining by whether the village is autonomous by ethnic minorities. (Yes = 1, No = 0)	Yes: 20.1%	No: 79.9%
Under 20	Continuous variable, referring to the proportion of villagers younger than 20 years old.	33.6%	2.7%
Over 65	Continuous variable, referring to the proportion of villagers older than 65 years old.	8.9%	0.9%
Economic income	Continuous variable, referring to the total gross output value of the village. (¥ 10,000)	9023.18	6791.91
Location	Continuous variable, referring to the road distance to the county government. (km)	28.03	13.72
Mountain village	Dummy variable, determining by whether the village is mountain village. (Yes = 1, No = 0)	Yes: 17.6%	No: 82.4%
**Town level**			
Professional equipment	Continuous variable, referring to the total value of high-quality medical. (¥ 10,000)	78.41	45.34
Healthcare bed	Continuous variable, referring to the actual number of healthcare beds.	63.33	30.56
Licensed doctor	Continuous variable, referring to the actual number of licensed doctors.	3.78	2.63
Nurse	Continuous variable, referring to the actual number of nurses.	10.93	9.72
Township population	Continuous variable, determining by the proportion of non-rural population in the town.	0.17	0.28
Aging degree	Continuous variable, determining by the proportion of the residential population older than 65 years old.	0.13	0.02
Industry structure	Continuous variable, determining by the proportion of agricultural output value in the total output value.	45.3%	33.9%
Public budget income	Continuous variable, referring to the public budget income of the town. ((¥ 10,000)	3935.82	5384.75

### Analysis methods

This study uses Gini coefficients, spatial autocorrelation analysis, multilevel regression model, and the difference in difference analysis. First, the temporal changes of PHC capacity are investigated by box plots and Gini coefficients, and the local spatial autocorrelation analysis is used to estimate the spatial disparities. The Gini coefficient is expressed as follows:

$$G=\frac{1}{2{n}^2\overline{x}}\ \sum_{i=1}^n\sum_{j=1}^n\mid {x}_i-{x}_j\mid$$

Where Gini coefficient (*G*) ranges from 0 (perfect equality) to 1 (perfect inequality); n refers to the number of villages included in the calculation; $\overline{x}$ is the average value of ‘Rural doctor’ or ‘Medical equipment’; ∣*x*_*i*_ − *x*_*j*_∣ denotes the absolute value of the differences between villages,

Second, the multilevel regression model is used to detect the impact of town- and village-level environments on the uneven allocation of rural doctors and medical equipment. The multilevel regression model is a widely used statistical method highlighting spatial heterogeneity, and it can examine the explanatory power of the independent variables by testing the change in variance in a set of models [40]. Setting the village level as the first level and the town level as the second level, we specify the model as follows:

1 $${\textrm{VHC}}_{\textrm{i}}={\upbeta}_1\cdot {\textrm{V}}_{\textrm{i}}+{\upbeta}_2\cdot {\textrm{T}}_{\textrm{j}}+{\textrm{y}}_{\textrm{i}}+{\upvarepsilon}_{\textrm{i}}$$

Where VHC_i_ is the allocation of primary healthcare resources in each administrative village; V_i_ and T_j_ represent the village-level and town-level environments, respectively; y_i_ is the time-fixed effect, and ɛ_i_ is the error term. The empty model’s intraclass correlation (ICC) is first used to examine whether a multilevel model is needed. Then, we sequentially include the village-level and town-level indicators to obtain the fitted model.

Third, given the complex relationship between township healthcare centers and villages in China, the benefits brought by recent policies to township healthcare centers may cause unexpected effects on the village institutions. Thus, to examine the dynamics of the driving mechanism of community environments on PHC inequalities, difference in difference analysis is used. According to the implementation of the PHC policy in Shuicheng, we set the four townships that first completed the reform in 2018 as the treated group and the other townships as the not-treated groups, with the samples in 2019 excluded. The conventional DID model is first conducted to examine the general policy intervention impact and we specify the model as follows:

2 $$VHC={\beta}_0+{\beta}_1\cdot \left({R}_i\cdot Polic{y}_{it}\right)+{\mu}_i+{\lambda}_t+\rho \cdot {X}_{it}+{\upvarepsilon}_{it}$$

Where *β*_*1*_ is the estimated coefficient denoting the policy intervention impact; *R*_*i*_ is a dummy variable that has a value of 1 if the township healthcare center has completed the construction and 0 otherwise; *Policy*_*it*_ is an indicative variable indicates whether the observed time spans the period after the policy; μ_i_ *and λ*_*t*_ refer to the individual fixed effect and time fixed effect. *X*_*it*_ represents other control variables; and *ɛ*_*it*_ is regression error term.

Then, the heterogeneous difference in difference model is used to examine whether the impact of town-level healthcare environment on village PHC capacity changes after the policy implementation. Rejecting the hypothesis that study objects in the experimental group own similar conditions, the heterogeneous difference in difference model enables scholars to examine the differentiated and nuanced outcomes that emerge after the policy intervention by setting additional interactive terms. In this study, we add a set of triple interactive terms based on the town-level healthcare characteristics (*TH*_*i*_), *R*_*i*_, and *Policy*_*it*_, and fit the model as:

3 $$VHC={\beta}_0+{\tau}_i\cdot {R}_i\cdot Polic{y}_{it}\cdot T{H}_{mi}+{\beta}_1\cdot \left({R}_i\cdot Polic{y}_{it}\right)+{\mu}_i+{\lambda}_t+\rho \cdot {X}_{it}+{\upvarepsilon}_{it}$$

Where *τ*_*i*_ is the key estimated coefficients denoting the heterogeneous effects; *TH*_*i*_ is the four town-level healthcare factors included in this analysis; other variables remain the same as above. Specifically, the precise effect size of each *TH*_*mi*_ should be interpreted as the sum of *τ*_*i*_ and *β*_*1*_, provided that *τ*_*i*_ is statistically significant.

Last, the robust test is conducted to check the reliability of our results, especially for the DID models (difference in difference). DID estimation is normally defined as valid based on a parallel trend assumption between the treatment group and the control group. Therefore, a graph plotting rural doctors per capita and medical equipment per capita for both groups before and after the ‘Yilianti’ reform is provided. Then, we conduct a falsification test by bringing forward the date of 1 year to create a false date of reform to refit the main regression in Eq. (2).

Data analysis is performed by StataMP 16, and a *p*-value less than 0.05 is considered statistically significant. Noted that some variables exhibit highly skewed distribution that could distort the relationships and significance tests, all the variables have been standardized with the Z-score method before data analysis. Therefore, the regression coefficient represents the average change in the dependent variable when the independent variable changes by one standard deviation. The largest VIF reported is 2.7.

## Research results

### The spatiotemporal variations of village-based PHC service capacity

**Fig. 3:**
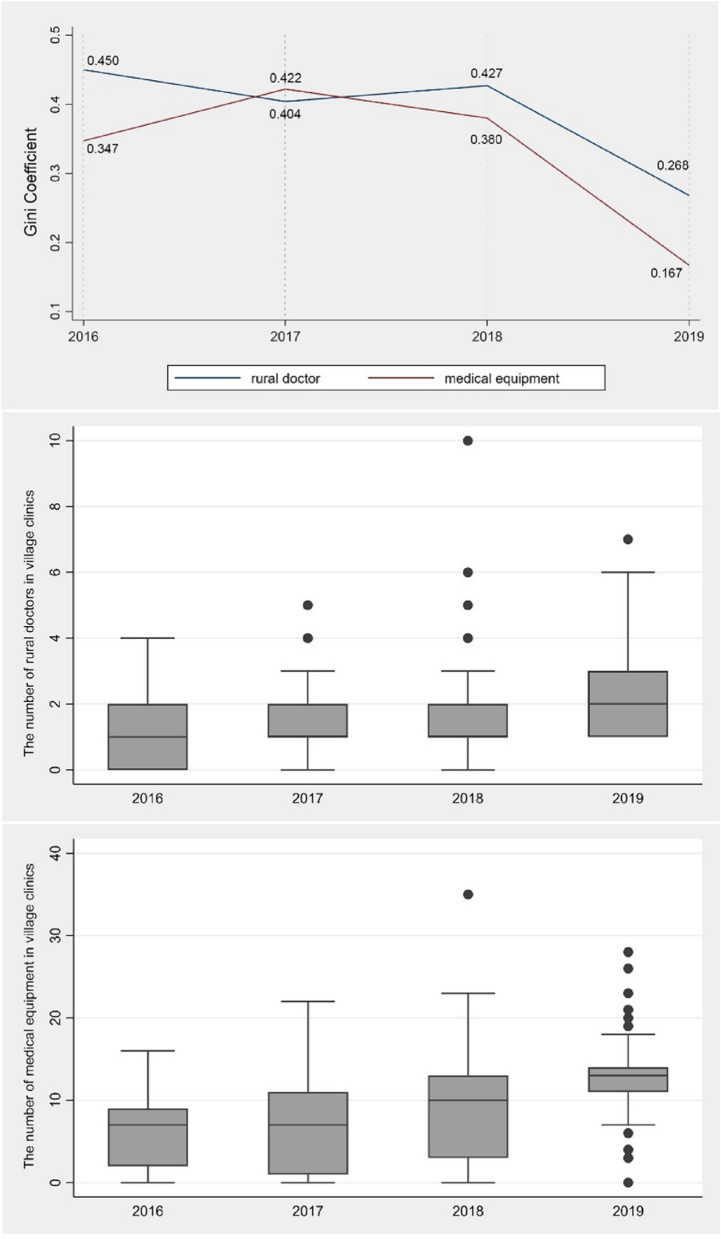
The temporal changes of village-level PHC inequalities from 2016 to 2019 (top: Gini coefficient; middle: the number of rural doctors per capita in clinics; bottom: the number of medical equipment per capita in clinics)

Figure 3 describes the temporal changes in village-level PHC service capacity during the study period. The Gini Coefficient for rural doctors and medical equipment in villages is 0.411 and 0.366, respectively, indicating that there are significant PHC inequalities across villages in Shuicheng County. The results suggest that the reform initiated in 2018 has greatly improved the uneven pattern of PHC delivery. There has been an annual increase in rural doctors per capita and medical equipment per capita from 2016 to 2019; however, the results also report an increased gap in the configuration level of rural doctors and a growth of villages that have seen a dramatic increase or decrease in the number of essential medical equipment per capita in their clinics.

**Fig. 4:**
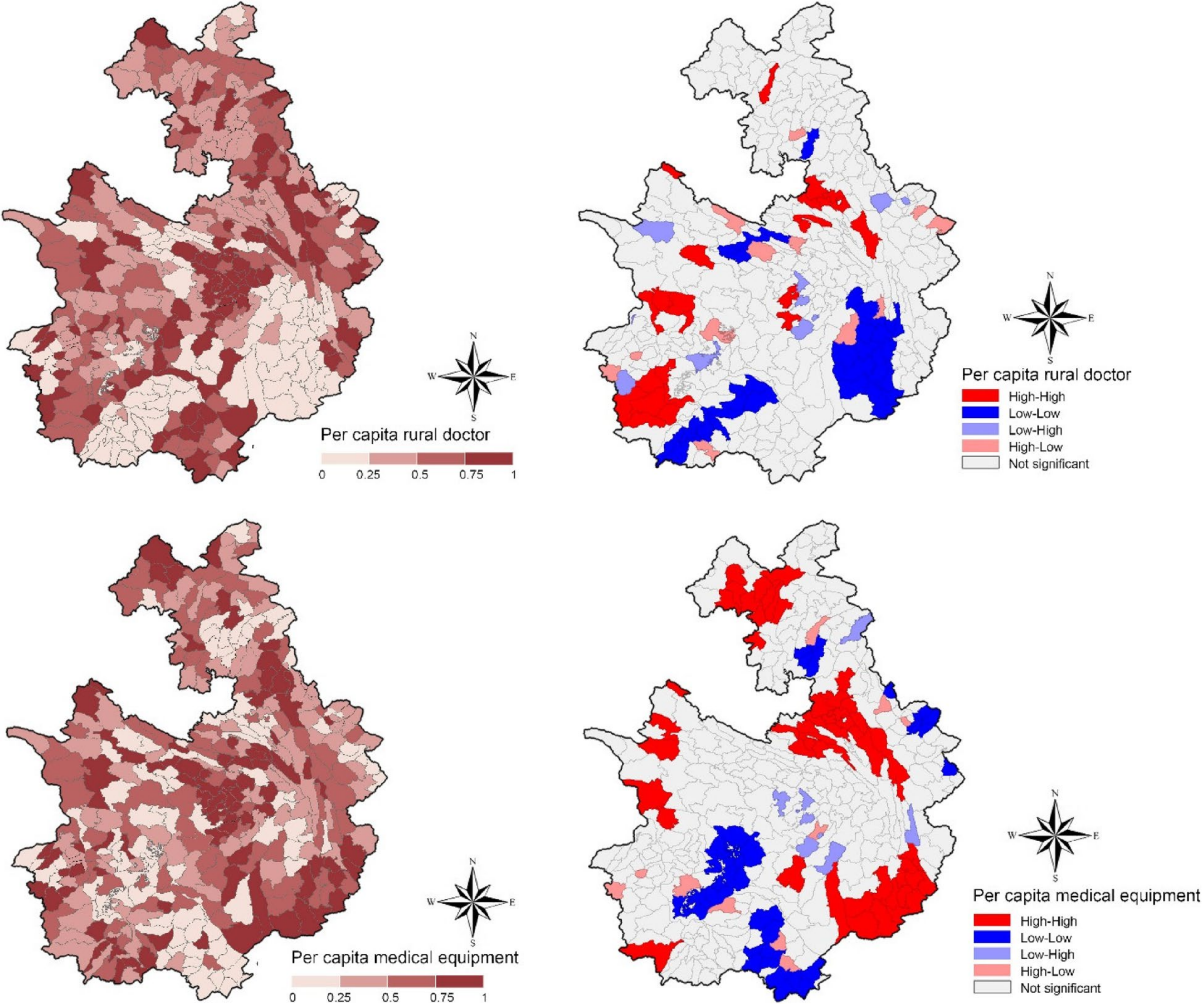
The spatial disparities and autocorrelation of rural doctors (top two pictures) and medical equipment (bottom two pictures) in Shuicheng county

Figure 4 depicts the spatial disparities and autocorrelations of village-level PHC service capacity. The PHCs in the central region of the county perform better in terms of both types of resources compared to other areas. It is worth noting that the spatial distribution baseline of rural doctors and medical equipment is not always consistent. For example, while the west-southern and eastern regions have a higher level of medical equipment configuration than other villages, the number of rural doctors per capita in these areas is relatively poor. Importantly, the results of local spatial autocorrelation analysis suggest a significant divergence in the clustering patterns of rural doctors and medical equipment calculated by the population, with only a few similar clusters overlapping.

### The driving effects of community environments on village-based PHC service capacity

The estimation results for the driving effects of community environments on village-level PHC capacity are shown in Table 2. For the configuration of rural doctors, the empty model reports that the ICC is 0.276, and the level 2 variance is significant (*p < 0.001*). Therefore, 27.6% of the total variation is caused by town-level differences, and a multilevel model is needed.

**Table 2:** Multilevel regression results of the associations between PHC capacity and community environments

	Rural doctor			Medical equipment		
	Empty model	Model (1)	Model (2)	Empty model	Model (3)	Model (4)
ICC	0.276***			0.397***		
**Village level**						
Ethnic autonomous		−0.122*** (0.048)	− 0.159** (0.048)		− 0.118*** (0.011)	− 0.112*** (0.010)
Under 20		−0.143*** (0.036)	−0.074*** (0.019)		− 0.099*** (0.009)	− 0.133*** (0.011)
Over 65		0.140*** (0.035)	0.069*** (0.017)		0.112*** (0.010)	0.144*** (0.012)
Economic income		0.018*** (0.005)	0.023** (0.006)		0. 075 (0.091)	0.070 (0.089)
Location		−0.070** (0.017)	− 0.067*** (0.017)		− 0.079* (0.008)	− 0.080* (0.008)
Mountain village		−0.086** (0.019)	− 0.080** (0.019)		− 0.063 (0.073)	− 0.061 (0.073)
**Town level**						
Professional equipment			0.012 (0.025)			0.015** (0.002)
Healthcare bed			0.099** (0.031)			−0.034 (0.044)
Licensed doctor			−0.057** (0.022)			−0.022*** (0.002)
Nurse			0.030** (0.008)			0.031*** (0.003)
Township population			−0.016 (0.031)			0.021** (0.002)
Aging degree			0.058** (0.021)			0.035 (0.078)
Industry structure			−0.052* (0.026)			−0.044 (0.052)
Public budget income			0.025 (0.023)			0.025 (0.031)
Time fixed effect	0.108*** (0.014)	0.092*** (0.016)	0.091*** (0.016)	0.260*** (0.018)	0.276*** (0.021)	0.307*** (0.029)
Cons	0.215*** (0.056)	0.426*** (0.084)	0.440*** (0.089)	1.471*** (0.094)	1.575*** (0.127)	1.471*** (0.135)
Random variance						
Intercept	0.063 (0.018)	0.061 (0.017)	0.054 (0.0.015)	0.214 (0.058)	0.201 (0.056)	0.188 (0.052)
Residual	0.164 (0.009)	0.160 (0.008)	0.153 (0.007)	0.325 (0.016)	0.317 (0.016)	0.314 (0.0.015)
- Loglikelihood	431.120	420.830	405.749	758.796	749.529	740.593
No. of obs.	946	946	946	946	946	946

(1) the results reported in the table are the coefficients, and the value of standard error is shown in parentheses. (2) ***, **, and * represent the 0.1, 1, and 5% significance levels, respectively

After adjusting all the variables in Model 2, the number of rural doctors per capita remains significantly associated with the local environment at both levels. For the village-level factors, the results of all explanatory variables are consistent with those in model 1. Generally, for the effect sizes, the demographic influences are more or less strong than the economic and locational factors. The number of rural doctors in ethnic minority autonomous villages is significantly less than that in non-minority autonomous areas. The result of ‘Over 65’ is positive, indicating that more rural doctors are clustered within aged villages. The result of ‘Under 20’, however, is the opposite. The result of ‘Location’ is negative and significant, that is, villages far away from the county center are disadvantaged in attracting rural doctors. Moreover, the results show that the number of rural doctors per capita in mountainous villages and impoverished villages is significantly less than in other areas.

Concerning the town-level environments, the impact of the township healthcare center is mixed. The results show that the linkages between agencies may have a more significant impact on rural doctors’ supply than administrative factors. Specifically, the result of ‘Healthcare bed’ is positive, indicating that the enhanced ability of township healthcare centers to receive inpatients may increase the subordinate village clinic’s competitiveness in attracting rural doctors. Similarly, the increase in nursing resources in township healthcare centers positively impacts the configuration of rural doctors. In contrast, the result of ‘Licensed doctor’ is negative and significant at the 0.001 level, indicating that the provision of rural doctors may decrease when the township healthcare centers decide to employ more licensed doctors. Additionally, the villages under the jurisdiction of towns with a high degree of aging and less dependence on agriculture are less likely to face a shortage of rural doctors.

On the other hand, in terms of the medical equipment, the empty model reports that the ICC is 0.397, and the level 2 variance is significant. With all the variables adjusted, the estimation results are reported in Model 4. At the village level, similar to the results for rural doctors, non-ethnic autonomous areas, villages with fewer young people, and more older people are likely to own more medical equipment. Clinics in remote villages may be disadvantaged in providing essential medical equipment to villagers.

The environmental factors at the town level are also significant, while the specific impact may be relatively weaker than their effects in the provision of rural doctors in clinics. The result of ‘Professional equipment’ indicates that township healthcare centers with more high-quality medical equipment could provide more support for subordinate villages in terms of hardware facilities. The result of ‘Nurse’ is similar. Consistent with the result for rural doctors, the effect of ‘Licensed doctor’ is adverse and indicates that the increase of professional staff in township healthcare centers would aggravate the shortage of medical equipment in villages. In addition, the urbanization process can benefit the growth of medical equipment in rural areas, perhaps owing to the increased financial ability.

### Effects of policy intervention

Table 3 reports the general policy intervention effects of the new healthcare reform on rural PHC. The result for medical equipment reports a positive impact of the recent reform (coef. = 0.350), that is, the increased mobility of medical resources within the county healthcare system can provide more support to the configuration of medical equipment in villages. However, the result of ‘Policy intervention’ for rural doctors is negative and significant (coef. = − 0.517), indicating that the policy of vertically integrating medical resources has further increased the shortage of rural doctors in villages. In other words, the rural regions that have completed the construction of the ‘county medical alliance’ have seen a faster decrease in the number of rural doctors per capita than those that have not. The results for community environments are similar to those in the previous models.

**Table 3:** The results of the DID regression models

	Rural doctor	Medical equipment
Policy intervention (β_1_)	−0.517** (0.199)	0.350* (0.198)
**Village level**		
Ethnic autonomous	−0.095*** (0.027)	−0.388*** (0.077)
Under 20	−0.120** (0.049)	0.068* (0.038)
Over 65	0.166*** (0.062)	0.109* (0.042)
Economic income	0.111*** (0.035)	0.088** (0.035)
Location	−0.043* (0.025)	−0.246*** (0.040)
Mountain village	−0.204*** (0.061)	−0.074 (0.061)
**Town level**		
Professional equipment	0.332*** (0.063)	0.312*** (0.063)
Healthcare bed	0.099* (0.044)	−0.089 (0.054)
Licensed doctor	−0.114** (0.042)	−0.164*** (0.042)
Nurse	0.072* (0.031)	0.064** (0.025)
Township population	−0.227*** (0.045)	−0.134** (0.044)
Aging degree	0.072* (0.034)	0.101** (0.034)
Industry structure	−0.057 (0.041)	−0.142** (0.041)
Public budget income	0.112* (0.046)	0.018 (0.045)
Cons	0.200*** (0.029)	0.223*** (0.018)
R square	0.328	0.395
No. of obs.	765	765

(1) the results reported in the table are the coefficients, and the value of standard error is shown in parentheses. (2) ***, **, and * represent the 0.1, 1, and 5% significance levels, respectively

By examining the heterogeneous effects of policy intervention, Table 4 reports the dynamics of the associations between community environments and PHC service capacity. The key exploratory variable is the interaction term ‘Policy intervention × Town-level healthcare (τ_m_).’ Collectively, although it is not always positive, the results show that the policy has further strengthened the impact of township PHC agencies on villages’ PHC service capacity. As indicated by the sum of β_1_ and τ_m_, i.e., − 0.475 + (− 0.039) = − 0.514, the inpatient service capacity of town healthcare centers may further accelerate the loss of rural doctors in villages after the policy. Similarly, the policy strengthens the negative impact of town healthcare centers recruiting licensed doctors and nurses on allocating rural doctors (the effect size is − 0.587 and − 0.344, respectively). On the other hand, the policy’s positive moderating effects on allocating medical equipment in villages may vary by the quality of the township healthcare centers. Both the results for ‘Professional equipment’ and ‘Nurse’ are significant and positive (the effect size is 0.504 and 0.184, respectively), indicating that the township healthcare centers with more professional equipment and nurses may more positively impact the clinic’s hardware resources after the policy.

**Table 4:** The results of the heterogeneous DID regression models

		Professional equipment	Healthcare bed	Licensed doctor	Nurse
Rural doctor	Policy intervention × Town-level healthcare (τ_m_)	− 0.102 (0.121)	− 0.475** (0.191)	− 0.541*** (0.106)	− 0.241* (0.116)
	Policy intervention (β_1_)	− 0.461** (0.209)	− 0.039 (0.298)	− 0.046 (0.224)	−0.103 (0.308)
Medical equipment	Policy intervention × Town-level healthcare (τ_m_)	0.340*** (0.089)	0.122 (0.191)	−0.084 (0.107)	0.231* (0.106)
	Policy intervention (β_1_)	0.164 (0.208)	0.359** (0.122)	0.437* (0.227)	−0.047 (0.307)
Village-level variables		Y	Y	Y	Y
Town-level variables		Y	Y	Y	Y
No. of obs.		745	745	745	745

(1) Other regression variables are the same as those in Table 3. They are omitted in the table for the sake of brevity (see Tables 5 and 6 in appendix). (2) the results reported in the table are the coefficients, and the value of standard error is shown in parentheses. (3) ***, **, and * represent the 0.1, 1, and 5% significance levels, respectively

### Robust test

**Fig. 5:**
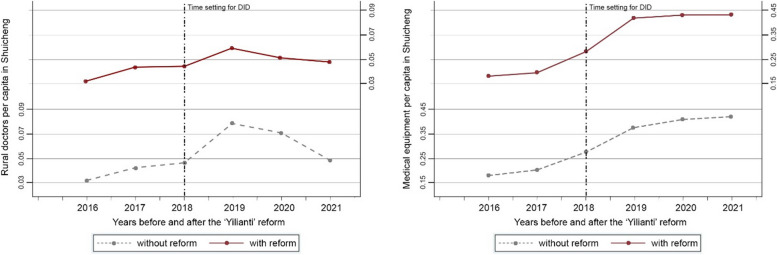
Parallel trend test of rural doctors per capita and medical equipment per capita before and after the ‘Yilianti’ reform. Notes: To complete a parallel trend test, we include the healthcare statistic data for 2020 and 2021. However, unfortunately, we could not obtain the other essential information needed in our regression models, e.g., information on township healthcare centers and town-level socio-economic conditions, mainly owing to the difficulties brought about by the COVID-19 pandemic. Therefore, the datasets used in our analysis are from 2016 to 2019

For the sake of conciseness, we present the results of robust test in the appendix. In the appendix, Fig. 5 shows the parallel trend test and Table 7 presents the falsification test. While the results pertaining to the independent variables at the rural and town levels are akin to those in Table 3, the outcomes concerning the critical explanatory variable, ‘Policy intervention,’ reveal no statistical significance. As such, the falsification test offers limited support for the notion of a spurious intervention impact of the primary healthcare policy on the allocation of PHC in villages. Consequently, our results are credible.

## Discussion

In recent years, healthcare inequalities have become increasingly prevalent in both the Global North and Global South, promoting scholars and policymakers to reconsider the uneven distribution of PHC resources through incorporating broader socioeconomic knowledge [2, 38, 41]. Focusing on the concept of community, the critical idea in the original conceptualization of PHC (WHO, 2021), this study systematically examines the nuanced and dynamic associations between community environments and the inequalities of PHC capacity in remote rural areas. Utilizing the facility-level healthcare statistical data in Shuicheng County during 2016 and 2019, the results of robustness confirm the emerged spatial autocorrelations in the distribution of PHC resources and reveal the driving forces of multiple community factors on PHC inequalities. The findings from this study help to enrich an interdisciplinary understanding of PHC inequalities by highlighting the place-based facility dimension of PHC delivery, and offer interdisciplinary suggestions to policymakers and planners. To our knowledge, this is the first micro-scale study examining the inequalities of PHC capacity through the lens of hierarchical community contexts in China’s remote rural areas.

### The spatial inequalities of village-level PHC service capacity

The pattern observed in this study captures the PHC allocation diversities among villages, complementing existing evidence of the uneven distribution of PHC resources in China at the micro-scale [20, 41]. Our results demonstrate that, although the latest healthcare reform has made significant strides in increasing primary healthcare capacity across locales [42], the uneven distribution of PHC resources, including rural doctors and medical equipment, remains significant. The geographically non-uniform patterns of various healthcare resources presented in this study are consistent with existing research that indicates the PHC disadvantages vary from place to place in different dimensions [24, 26]. Moreover, echoing the knowledge of the social determinants in healthcare inequalities [8], the spatial correlations results show that the villages with similar PHC capacity seem to cluster in specific locations, i.e., high-high pattern or low-low pattern, suggesting that the inequalities of PHC capacity could be spatially interrelated and associated with place-based socioeconomic characteristics.

### Driving mechanism regarding multilevel community environments

Using the multilevel regression model, this study reveals the nuanced associations between PHC capacity and community environment, which helps to interpret the long-standing supply-side inequalities of rural primary healthcare delivery [38, 39]. For the localized healthcare demands, consistent with X. Wang and Pan’s [18] findings, ethnic minority autonomous areas face a disadvantaged position concerning rural primary healthcare services. Other village contexts, including demographics and location, have significant effects on the configuration of rural doctors and medical equipment. Perhaps due to the critical position of the elderly in the current PHC system, villages with a higher degree of aging are more likely to have better healthcare services. However, an aging-oriented configuring mechanism may result in young people being neglected in the organizing process, adding a new dimension to the usually observed age-related healthcare inequality in China [43]. This study also finds that villages far away from the county center, particularly in mountainous areas, are less likely to have more healthcare resources, supplementing existing literature that emphasizes how rural areas with weak economic development often become less attractive labor markets [17]. Regarding town-level administrative settings, township population concentration positively affects the configuration of medical equipment, but it may also lead to a loss of rural doctors. The impact of aging degree on PHC is similar to that at the village level, while an inertia dependence on traditional agriculture may negatively impact the configuration of rural doctors.

Noting the institutional nature of PHC agencies in rural China, our findings note the impact of township healthcare centers on village PHC capacity in two ways. Our study reveals that although the town-level PHC delivery can positively influence the allocation of medical equipment and rural doctors in villages, the recruitment of licensed doctors in township healthcare centers may decrease the attractiveness of villages to rural doctors. These findings indicate that despite the provision of financial support and medical staff training by township healthcare centers with better healthcare and economic conditions, there remains a significant competition for medical resources, particularly professional labor, between town and village healthcare facilities. A possible explanation is the overall shortage and the promoting mechanism of rural doctors, and the competition of various stakeholders in the primary healthcare market (Zhang and Zhou 2018 [26];). To this extent, the influences of township healthcare centers on village clinics could be understood as a social dimension of community contexts rather than merely institutional forces within the healthcare system.

### The policy intervention impact

Employing the difference-in-difference analysis with two-way fixed effects to examine the intervention impact of policy, this study reveals the dynamics of the relationship between PHC supply-side inequalities and community environments, especially the town-level PHC agencies. Echoing the current debates [24, 26], the enhancement of town-level healthcare would inevitably cause effects on villages, although the effects are not always conducive. While increasing medical equipment, constructing a ‘county medical alliance’ may also accelerate the loss of rural doctors in specific villages. Furthermore, the intervention impact on villages varies by the township healthcare landscape. The new round of reform may strengthen the negative impact of township healthcare centers on villages’ attractiveness to rural doctors, interpreted as the increased career development opportunities in township centers due to the encouraged healthcare assistance from county hospitals, including telemedicine assistance and the dispatch of high-level doctors [44]. On the other hand, after the policy implementation, township healthcare centers with more professional equipment and nurses may contribute to further improving clinics’ hardware settings. These findings offer novel and nuanced understandings of the complex associations between towns’ and villages’ PHC capacity, while the detailed mechanism underlining these changes requires further investigation.

### Policy implications

Our research has significant policy implications for China’s healthcare reform. First, in addition to demographic-based allocation mechanisms, policymakers must consider multiple community contexts when making planning. Our study reveals that various factors, such as localized healthcare needs, institutional interaction between various agencies, and administrative features in China, can significantly impact the inequalities of PHC service capacity. Given that villagers in disadvantaged areas are usually exposed to higher health risks [20, 43], policymakers should adopt a multi-dimensional approach to allocating PHC resources, to avoid exacerbating existing health inequalities. Second, our study emphasizes the need for a town-village lens in PHC governance. Over the past two decades, the market-oriented transformation of China’s healthcare system has created a profit-making attribute for township healthcare centers, leading to a complex relationship with village-level PHC services. With the increasing trend of township-centric PHC reform and revocation and merger of administrative villages (‘合村并居’ in Chinese), policymakers must develop policies that promote coordination between town- and village-level healthcare institutions. Third, policymakers must be cautious about the potential side effects of current healthcare reform. While villagers may benefit from enhanced PHC quality in township healthcare centers, there could be unexpected external effects on villages, leading to new healthcare disparities and inequalities across rural settings.

## Conclusion

To summarize, by underlining the place-based facility dimension of PHC delivery, this study enriches the understanding of how community contexts contribute to the widening spatial inequalities in the rural primary healthcare configuration process. Despite the awareness of the wide mismatch of supply and demand in PHC, little is known about the dynamic relationship between the community environments and the inequalities in PHC service capacity. Addressing this long-term limitation in healthcare research, the interdisciplinary knowledge, e.g., ‘geographical lens’, is increasingly recognized as a valuable and reliable approach due to its ability to capture the nuanced relationships between local environments and healthcare delivery [3]. Responding to the critical challenges in extent studies, this study is novel in that it uses a four-year hospital group from remote rural areas to granularly and dynamically understand how rural primary healthcare allocation varies by local environments at both town and village levels. On the one hand, this study proves and extends the impact of broader community socio-spatial characteristics on PHC configuration; on the other hand, the more accurate assessment of PHC service offers reliable evidence about the place-based understanding of PHC delivery in health geography. Confirming the spatial correlations of the disparities of PHC service capacity, we also reveal the mixed intervention impact of recent healthcare policies on village-level PHC services, thus contributing to a supply-side dynamic understanding of the mutual relationship between the community and resource configuration. The findings from this study suggest that broad community environments, including the physical, social, and economic attributes and the institutional structures, may influence the (re)configuration process of rural primary healthcare through causal pathways that warrant further investigation in future studies.

## Study limitation

We acknowledge some limitations of this study, mainly related to data availability. First, due to the lack of geographic information on the specific residences, we use town and village council locations to represent the geographical coordinates of villagers. This assumption of uniform geographic distribution may lead to a simplified description of the actual community environment. Second, despite access to official statistical documents, some key information is still lacking, including gender ratio, average income level of villagers, and average education level. Moreover, although there are some other types of labels about community’s economic and social characteristics in statistical yearbook, their data are missing to a considerable extent in different years, possibly due to the greater difficulty of statistical work in remote villages. This greatly limits the selection of corresponding independent variables in the study. Lastly, based on the associations revealed by quantitative methods in this study, qualitative research will undoubtedly contribute to a deeper understanding of community orientation in primary healthcare configuration by interviewing villagers and officials. These limitations will be addressed in the future through more empirical studies.

## Data Availability

The datasets generated or analyzed during this study are available from the corresponding author on reasonable request.

## Appendix

The 12 kinds of essential medical equipment configured in each village clinic include: respirator, sphygmomanometers, stethoscopes, refrigerated box, sterile cabinet, traditional Chinese medicine cabinet, western medicine cabinet, medical stretcher, medical disposal platform, integrated healthcare machine, computer, and printer.

**Table 5:** The results of the heterogeneous DID regression models by the number of rural doctors per capita

	Rural doctor			
	Professional equipment	Healthcare bed	Licensed doctor	Nurse
Policy intervention × Town-level healthcare (τ_m_)	− 0.102 (0.121)	− 0.475** (0.191)	− 0.541*** (0.106)	− 0.241* (0.116)
Policy intervention (β_1_)	− 0.461** (0.209)	− 0.039 (0.298)	− 0.046 (0.224)	− 0.103 (0.308)
**Village level**				
Ethnic autonomous	−0.093*** (0.027)	−0.085*** (0.025)	− 0.079*** (0.025)	− 0.099*** (0.027)
Under 20	− 0.117** (0.049)	− 0.109** (0.049)	− 0.099** (0.048)	− 0.112** (0.049)
Over 65	0.163*** (0.062)	0.154*** (0.062)	0.147*** (0.061)	0.157*** (0.062)
Economic income	0.108*** (0.035)	0.102*** (0.035)	0.102*** (0.035)	0.105*** (0.035)
Location	−0.045* (0.025)	−0.045* (0.025)	− 0.042* (0.025)	−0.049* (0.026)
Mountain village	−0.204*** (0.061)	−0.202*** (0.061)	− 0.206*** (0.060)	−0.200*** (0.061)
**Town level**				
Professional equipment	0.365*** (0.074)	0.379*** (0.065)	0.432*** (0.065)	0.385*** (0.069)
Healthcare bed	0.104* (0.045)	0.087* (0.041)	0.086* (0.041)	0.107* (0.045)
Licensed doctor	−0.110** (0.042)	−0.095** (0.041)	− 0.102** (0.042)	−0.111** (0.042)
Nurse	0.077* (0.031)	0.080* (0.032)	0.075* (0.031)	0.077* (0.031)
Township population	−0.237*** (0.046)	− 0.253*** (0.046)	− 0.257*** (0.046)	−0.246*** (0.046)
Aging degree	0.070* (0.034)	0.073* (0.034)	0.075* (0.034)	0.066* (0.034)
Industry structure	−0.070 (0.041)	− 0.043 (0.039)	− 0.065 (0.041)	− 0.056 (0.041)
Public budget income	0.104* (0.045)	0.123** (0.047)	0.114* (0.046)	0.118* (0.046)
Cons	0.217*** (0.030)	0.225*** (0.031)	0.206*** (0.029)	0.213*** (0.030)
R square	0.327	0.334	0.326	0.330
No. of obs.	765	765	765	765

***, **, and * represent the 0.1, 1, and 5% significance levels, respectively

**Table 6:** The results of the heterogeneous DID regression models by the number of medical equipment per capita

	Medical equipment			
	Professional equipment	Healthcare bed	Licensed doctor	Nurse
Policy intervention × Town-level healthcare (τ_m_)	0.340*** (0.089)	0.122 (0.191)	−0.084 (0.107)	0.231* (0.106)
Policy intervention (β_1_)	0.164 (0.208)	0.359** (0.122)	0.437* (0.227)	−0.047 (0.307)
**Village level**				
Ethnic autonomous	−0.393*** (0.077)	−0.391*** (0.077)	− 0.377*** (0.075)	−0.384*** (0.077)
Under 20	0.067* (0.038)	0.065* (0.038)	0.072* (0.039)	0.061 (0.049)
Over 65	0.108* (0.042)	0.106* (0.042)	0.112* (0.042)	0.101 (0.062)
Economic income	0.098** (0.036)	0.091** (0.035)	0.087** (0.035)	0.094** (0.035)
Location	−0.239*** (0.040)	−0.246*** (0.040)	− 0.245*** (0.040)	−0.241*** (0.040)
Mountain village	−0.074 (0.060)	−0.073 (0.061)	− 0.073 (0.061)	−0.071 (0.061)
**Town level**				
Professional equipment	0.204*** (0.037)	0.301*** (0.062)	0.328*** (0.066)	0.262*** (0.053)
Healthcare bed	−0.106* (0.054)	−0.086 (0.054)	− 0.090 (0.054)	−0.097 (0.054)
Licensed doctor	−0.177*** (0.042)	−0.168*** (0.042)	− 0.163*** (0.042)	−0.166*** (0.042)
Nurse	0.062** (0.025)	0.066** (0.025)	0.066** (0.025)	0.089 (0.062)
Township population	−0.100** (0.032)	−0.128** (0.044)	− 0.144** (0.046)	−0.115* (0.046)
Aging degree	0.095** (0.034)	0.101** (0.034)	0.100** (0.034)	0.096** (0.034)
Industry structure	−0.150** (0.041)	−0.146** (0.041)	− 0.135** (0.041)	−0.143** (0.041)
Public budget income	0.029 (0.048)	0.011 (0.047)	0.030 (0.048)	0.007 (0.048)
Professional equipment	0.225*** (0.018)	0.231*** (0.019)	0.213*** (0.017)	0.229*** (0.018)
R square	0.393	0.397	0.395	0.396
No. of obs.	765	765	765	765

***, **, and * represent the 0.1, 1, and 5% significance levels, respectively

**Table 7:** Falsification test

	Rural doctor	Medical equipment
Policy intervention (β_1_)	−0.369 (0.295)	0.083 (0.195)
**Village level**		
Ethnic autonomous	−0.082** (0.024)	−0.376*** (0.078)
Under 20	−0.122** (0.047)	0.123** (0.047)
Over 65	0.178*** (0.058)	0.184* (0.058)
Economic income	0.111*** (0.035)	0.089** (0.035)
Location	−0.040 (0.041)	−0.237*** (0.041)
Mountain village	−0.218*** (0.061)	−0.079 (0.061)
**Town level**		
Professional equipment	0.334*** (0.063)	0.315*** (0.063)
Healthcare bed	0.117* (0.054)	−0.107* (0.055)
Licensed doctor	−0.119** (0.042)	−0.173*** (0.042)
Nurse	0.061 (0.067)	0.055 (0.067)
Township population	−0.206*** (0.042)	−0.191** (0.042)
Aging degree	0.065 (0.037)	0.129** (0.037)
Industry structure	−0.062 (0.041)	−0.091** (0.041)
Public budget income	0.099* (0.043)	0.099* (0.043)
Cons	0.146*** (0.023)	0.283*** (0.038)
R square	0.313	0.373
No. of obs.	765	765

***, **, and * represent the 0.1, 1, and 5% significance levels, respectively

## References

[1] Organization, World Health (1978). Declaration of alma-ata.

[2] Sanders D, Nandi S, Labonté R, Vance C, Van Damme W (2019). From primary health care to universal health coverage—one step forward and two steps back. Lancet.

[3] Crooks VA, Andrews GJ (2009). Community, equity, access: core geographic concepts in primary health care. Prim Health Care Res Dev.

[4] Harris SB, Green ME, Brown JB, Roberts S, Russell G, Fournie M, Webster-Bogaert S, Paquette-Warren J, Kotecha J, Han H (2015). Impact of a quality improvement program on primary healthcare in Canada: a mixed-method evaluation. Health Policy.

[5] Hone T, Macinko J, Millett C (2018). Revisiting Alma-Ata: what is the role of primary health care in achieving the sustainable development goals?. Lancet.

[6] King R, Green P (2012). Governance of primary healthcare practices: Australian insights. Bus Horiz.

[7] Lewin S, Lavis JN, Oxman AD, Bastías G, Chopra M, Ciapponi A, Flottorp S, Martí SG, Pantoja T, Rada G (2008). Supporting the delivery of cost-effective interventions in primary health-care systems in low-income and middle-income countries: an overview of systematic reviews. Lancet.

[8] Owusu L, Yeboah T (2018). Living conditions and social determinants of healthcare inequities affecting female migrants in Ghana. GeoJournal.

[9] Bulmer M (1986). The Chicago school of sociology: institutionalization, diversity, and the rise of sociological research.

[10] Park RE. Human communities: The city and human ecology. Free Press, London: Collier-Macmillan; 1968.

[11] Greer SA (1962). The emerging city: myth and reality.

[12] Keller SI (1968). The urban neighborhood: a sociological perspective. Vol. 33.

[13] Schwirian KP (1983). Models of neighborhood change. Annu Rev Sociol.

[14] WHO. “Primary Health Care.” World Health Organization, April 1, 2021. https://www.who.int/news-room/factsheets/detail/primary-health-care. visit time: 3 June 2023.

[15] Bath J, Wakerman J (2015). Impact of community participation in primary health care: what is the evidence?. Aust J Prim Health.

[16] Haggerty J, Burge F, Lévesque JF, Gass D, Pineault R, Beaulieu MD, Santor D (2007). Operational definitions of attributes of primary health care: consensus among Canadian experts. Ann Fam Med.

[17] Amin A, Dutta M, Mohan SB, Mohan P. Pathways to enable primary healthcare nurses in providing comprehensive primary healthcare to rural, tribal communities in Rajasthan, India. Front Public Health. 2020;831 10.3389/fpubh.2020.583821 PMC7728734 33330325

[18] Wang X, Pan J (2016). Assessing the disparity in spatial access to hospital care in ethnic minority region in Sichuan Province, China. BMC Health Serv Res.

[19] Filho S, Martins A, Vasconcelos CH, Dias AC, de Souza ACC, Merchan-Hamann E, da Silva MRF (2022). Primary health Care in Northern and Northeastern Brazil: mapping team distribution disparities. Ciênc Saúde Colet.

[20] Wang S, Jin X, Jiang X, Li C, Li H, Song S, Huang E, Meng Q (2018). Trends in health resource disparities in primary health care institutions in Liaoning Province in Northeast China. Int J Equity Health.

[21] McGrail MR, Humphreys JS. Measuring spatial accessibility to primary health care services: Utilising dynamic catchment sizes. Appl Geogr. 2014;54:182–8.

[22] Langford M, Higgs G. Measuring potential access to primary healthcare services: the influence of alternative spatial representations of population. The Professional Geographer. 2006;58(3):294-306.

[23] Kotavaara O, Nivala A, Lankila T, Huotari T, Delmelle E, Antikainen H. Geographical accessibility to primary health care in Finland–Grid-based multimodal assessment. Appl Geogr. 2021;136:102583.

[24] Ao Y, Feng Q, Zhou Z, Chen Y, Wang T (2022). Resource allocation equity in the China’s rural three-tier healthcare system. Int J Environ Res Public Health.

[25] Li X, Jiapeng L, Shuang Hu KK, De Cheng J, Maeseneer QM, Mossialos E, Dong Roman X, Yip W, Zhang H (2017). The primary health-care system in China. Lancet.

[26] Chen Y, Sylvia S, Paiou W, Yi H (2022). Explaining the declining utilization of village clinics in rural China over time: a decomposition approach. Soc Sci Med.

[27] Zhang JC, DY Z. "Analysis on the current status, problems and countermeasures of medical service capabilities in the Chinese village clinics." Chin J Health Policy. 2018;11(7):67-72.

[28] General Office of the State Council in China. (2009, March 17). Opinions on Deepening and Promoting the Reform of the Medical and Health System. Central People’s Government of the People’s Republic of China. https://www.gov.cn/gongbao/content/2009/content_1284372.htm. Accessed 3 June 2023.

[29] Liupanshui Municipal Government Office. (2016, December 30). Notice from the Municipal People’s Government Office on Issuing the Three-Year Plan for Improving Primary Medical and Health Service Capacity in Liupanshui City (2016-2018). Liupanshui Municipal People’s Government Website. https://zfgb.gzlps.gov.cn/zfgb/2017nd2q/swbszfbwj_49828/201705/t20170508_12823561.html. Accessed 3 June 2023.

[30] Chinese Civilization Network. (2017, March 1). Shuicheng County Launches Integrated Medical and Health Management Mechanism. Guizhou News. http://jiangsu.china.com.cn/html/2017/gznews_0301/9569937.html. Accessed 8 June 2023.

[31] Shuicheng County Party Committee Propaganda Department. (2018, May 16). Shuicheng County: Launching a Combination of Health and Poverty Alleviation to Fully Launch the Construction of a “County-Level Medical Alliance.” Digital Guizhou. http://wsgz.gog.cn/system/2018/05/16/016587852.shtml. Accessed 8 June 2023.

[32] Liupanshui City Health Bureau. 2018a. On-site promotion meeting of Liupanshui medical consortium in Shuicheng County. July 10, 2018. http://swjj.gzlps.gov.cn/gzdt/201807/t20180710_13070176.html

[33] Liupanshui Municipal Government. 2022. The 2020 Shuicheng County government work report. November 27, 2022. http://zgcounty.com/news/17092.html

[34] Kamel Boulos, Maged N, and Estella M Geraghty. 2020. Geographical tracking and mapping of coronavirus disease COVID-19/severe acute respiratory syndrome coronavirus 2 (SARS-CoV-2) epidemic and associated events around the world: how 21st century GIS technologies are supporting the global fight against outbreaks and epidemics. Springer. 10.1186/s12942-020-00202-8 PMC7065369 32160889

[35] Nethery RC, Rushovich T, Peterson E, Chen JT, Waterman PD, Krieger N, Waller L, Coull BA (2021). Comparing denominator sources for real-time disease incidence modeling: American community survey and WorldPop. SSM-Popul Health.

[36] Ouko JJO, Gachari MK, Sichangi AW, Alegana V. Geographic information system‐based evaluation of spatial accessibility to maternal health facilities in Siaya County, Kenya. Geogr Res. 2019;57(3):286–98.

[37] Xu X. “China GDP spatial distribution kilometer grid data set.” Data Registration and publishing System of Resources and Environmental Sciences Data Center, Chinese Academy of Sciences. 2017;10.

[38] Danish A, Blais R, Champagne F. Strategic analysis of interventions to reduce physician shortages in rural regions. Rural Remote Health. 2019;19(4) 10.22605/RRH5466 31752495

[39] Sharma DC (2015). India still struggles with rural doctor shortages. Lancet.

[40] Austin PC, Merlo J (2017). Intermediate and advanced topics in multilevel logistic regression analysis. Stat Med.

[41] Wang X, Yang H, Duan Z, Pan J (2018). Spatial accessibility of primary health care in China: a case study in Sichuan Province. Soc Sci Med.

[42] Yuan S, Fan F, Zhu D (2022). Effects of vertical integration reform on primary healthcare institutions in China: evidence from a longitudinal study. Int J Health Policy Manag.

[43] Fu L, Fang Y’n, Dong Y (2022). The healthcare inequality among middle-aged and older adults in China: a comparative analysis between the full samples and the homogeneous population. Heal Econ Rev.

[44] Shen S (2022). Continue to promote the construction of county medical community.

